# Elevated HbA1c Levels Are Associated with a Risk of Pancreatic Cancer: A Case–Control Study

**DOI:** 10.3390/jcm13185584

**Published:** 2024-09-20

**Authors:** Steven Grewe, Markus S. Jördens, Christoph Roderburg, Catherine Leyh, Simon Labuhn, Tom Luedde, Sarah Krieg, Andreas Krieg, Sven H. Loosen, Karel Kostev

**Affiliations:** 1 Department of Gastroenterology, Hepatology and Infectious Diseases, University Hospital Düsseldorf, Medical Faculty, Heinrich Heine University Düsseldorf, Moorenstrasse 5, 40225 Düsseldorf, Germany; steven.grewe@med.uni-duesseldorf.de (S.G.); markus.joerdens@med.uni-duesseldorf.de (M.S.J.); catherine.leyh@med.uni-duesseldorf.de (C.L.); simon.labuhn@med.uni-duesseldorf.de (S.L.); tom.luedde@hhu.de (T.L.); sven.loosen@med.uni-duesseldorf.de (S.H.L.); 2 Department of Inclusive Medicine, University Hospital Ostwestfalen-Lippe, Bielefeld University, 33617 Bielefeld, Germany; sarah.krieg@mara.de; 3 Department of General and Visceral Surgery, Thoracic Surgery and Proctology, University Hospital Herford, Medical Campus OWL, Ruhr University Bochum, 32049 Herford, Germany; andreas.krieg@klinikum-herford.de; 4 Epidemiology, IQVIA, 60549 Frankfurt, Germany

**Keywords:** pancreatic cancer, PDAC, Hba1c, early detection

## Abstract

**Background:** The early diagnosis of pancreatic cancer (ICD-10 C25) can improve the patient’s prognosis. The association between pancreatic cancer and type 2 diabetes (T2D) is known, but not yet fully understood. It is, therefore, necessary to investigate the impact of hemoglobin A1c (HbA1c) serum levels on pancreatic cancer development and the potential intervention options. **Methods:** In the case–control study, patients from the German IQVIA^TM^ Disease Analyzer database aged ≥18 years with a diagnosis of pancreatic cancer (ICD-10 C25) and a diagnosis of T2D (ICD-10: E11) were included. The patients’ propensity score matched 1:5 with individuals without pancreatic cancer. Logistic regression models were used to estimate odds ratios (ORs) with 95% confidence intervals (95% CI). **Results:** An elevated serum HbA1c prior to the index date was found to be significantly associated with an increased risk of a subsequent pancreatic cancer diagnosis for the mean HbA1c values of 6.5–8.4% (OR: 1.38; 95% CI: 1.22–1.57) as well as for mean HbA1c values ≥8.5% (OR: 1.41; 95% CI: 1.16–1.73). The only antihyperglycemic agent negatively associated with the subsequent pancreatic cancer diagnosis was the sodium–glucose cotransporter-2 (SGLT-2) inhibitor, with an odds ratio of 0.80 (95% confidence interval: 0.74–0.87 per year of therapy). This correlation was observed in both age- and sex-stratified subgroups. **Conclusions:** The data indicate that elevated serum HbA1c levels in patients with T2D are associated with an increased risk of pancreatic cancer development. It is possible that SGLT2 therapy may prove an effective means of reducing the risk of pancreatic cancer, thereby offering a potential avenue for the future reduction in pancreatic cancer incidence in patients with T2D.

## 1. Introduction

Pancreatic cancer (ICD-10 C25) is a highly aggressive tumor with a poor prognosis, reflected in a 5-year overall survival rate of approximately 10% [1,2].

In 2020, 495,773 new cases and 466,003 deaths were reported worldwide [3], with most initial diagnoses occurring at an advanced stage [4]. Late diagnosis is mainly due to the delayed onset of symptoms, and systemic treatment options are very limited. Therefore, the identification of efficient, simple, and minimally invasive predictors for the early diagnosis of pancreatic cancer is crucial to increase the chances of curative treatment in high-risk patients.

Diabetes, which affected approximately 529 million people worldwide in 2021 [5], has been linked to the development of pancreatic cancer [6]. Elevated insulin and glucose levels may increase the risk of malignant transformation and the development of pancreatic cancer [7]. Approximately 1% of adults with new-onset type 2 diabetes (T2D) will develop pancreatic cancer within three years [8], highlighting the importance of the effective management of T2D in these patients. In 74–88% of patients with an initial diagnosis of pancreatic ductal adenocarcinoma, T2D had been diagnosed within the previous 24 months [9].

Therefore, this study aimed to investigate the association of serum haemoglobinA1c (HbA1c) levels and the subsequent pancreatic cancer in patients with T2D. The study also investigated whether antihyperglycemic agents are associated with a risk of pancreatic cancer.

## 2. Methods

### 2.1. Database

This study used data from German primary care practices from the Disease Analyzer database (owned by IQVIA). Details of the methodology have been published previously [10]. In brief, the Disease Analyzer database contains data on demographic variables, diagnoses, and prescriptions from general and specialist practices in Germany. Practices included in the database are selected according to the annual statistics of the German Medical Association, which include information on the age of the physician, specialty group, community size category, and federal state. The database includes approximately 1300 general practices in Germany. The panel of practices included in the Disease Analyzer database has previously been shown to be representative of the general and specialist practices in Germany [10].

### 2.2. Study Population

**Figure 1:**
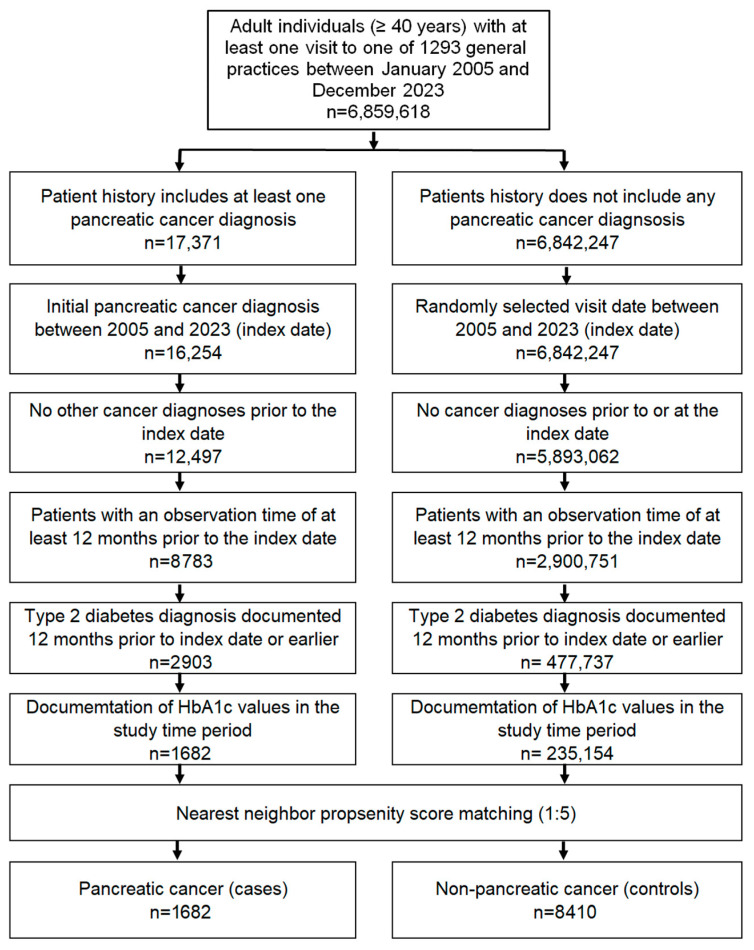
Selection of study patients.

The study population included all the patients aged ≥18 years with an initial pancreatic cancer diagnosis (ICD-10 C25) between January 2005 and December 2023 (index date), with at least one year of follow-up before the index date and a diagnosis of T2D (ICD-10: E11) at least 12 months before the index date. The controls were T2D individuals without cancer matched (1:5) using the nearest neighbor propensity scores based on age, sex, duration of diabetes, and diagnoses documented within five years prior to the index date, including obesity (ICD-10: E66); hypertension (ICD-10: E10); dyslipidemia (ICD-10: E78); pancreatic diseases (ICD-10: K85-K87); diseases of the esophagus, stomach, and duodenum (ICD-10: K20-K31); and chronic kidney disease (ICD-10:N18, N19). For individuals without cancer, the index date was a randomly selected visit date between January 2005 and December 2023. The flow diagram of the study participants is shown in Figure 1.

### 2.3. Study Objectives

The aim of the study was to investigate the association between serum HbA1c levels, antihyperglycemic therapy, and subsequent pancreatic cancer. All the HbA1c serum levels documented from six months before the first diabetes diagnosis and the index date were considered and an average value was calculated. The mean HbA1c serum level was classified as reference (HbA1c < 6.5%), elevated (6–5–8.4%), and severely elevated (≥8.5%). Additionally, all the serum fasting glucose levels documented from six months before the first diabetes diagnosis and the index date were considered and an average value was calculated. The mean fasting glucose level was classified as reference (<125 mg/dL), elevated (125–150 mg/dL), and severely elevated (>150 mg/dL%).

The duration of antihyperglycemic medication prescribed between diabetes diagnosis and the index date was also calculated in years. These drugs included metformin, sulfonylureas, dipeptidyl peptidase-4 (DPP-4) inhibitors, glucagon-like peptide-1 (GLP-1) receptor agonists, sodium–glucose transporter-2 (SGLT-2 inhibitors), and insulin.

### 2.4. Statistical Analyses

The demographic and clinical characteristics of the cases and controls after 1:5 propensity score matching were assessed using the standardized mean difference (SMD), which is considered the most commonly used statistic to examine the balance of the covariate distribution between treatment groups. An SMD of less than 0.1 was allowed, indicating that adequate covariate balance was achieved [11].

To examine whether HbA1c, fasting glucose, and antihyperglycemic therapy were associated with the subsequent pancreatic cancer diagnosis, we used multivariable conditional logistic regression models and estimated odds ratios (ORs) with 95% confidence intervals (95% CI). All the models were also calculated separately for women and men, and for three age groups. To allow for multiple comparisons, a *p*-value < 0.01 was considered statistically significant. All the analyses were performed using SAS version 9.4 (SAS Institute, Cary, NC, USA).

## 3. Results

### 3.1. Baseline Characteristics

After a 1:5 matching, 1682 cases (individuals with pancreatic cancer) and 8410 controls (individuals without pancreatic cancer) were available for analysis. The mean age at the index date was 74.4–74.5 years and 47% were female. On average, both the cases and controls had had diabetes for 6.8 years prior to the index date. The prevalence of predefined co-diagnoses was similar between the cases and controls (all standardized mean differences < 0.1) (Table 1). The mean number of documented HbA1c serum levels was 14.5 in the cases and 13.5 in the controls (Table 2).

### 3.2. Association between HbA1c Values and Subsequent Pancreatic Cancer Diagnosis

The average HbA1c value was higher in the cases (7.0%; SD: 1.1) than in the controls (6.8%; SD: 1.1). The elevated average HbA1c values prior to the index date were found to be significantly associated with an increased risk of subsequent pancreatic cancer for the average HbA1c values of 6.5–8.4% (OR: 1.38; 95% CI: 1.22–1.57) as well as for the average HbA1c values of ≥8.5% (OR: 1.41; 95% CI: 1.16–1.73). In the age- and sex-stratified analysis, an average HbA1c value between 6.5 and 8.4% was found to be significantly associated with an increased risk of pancreatic cancer in women (OR: 1.31; 95% CI: 1.07–1.61) and men (OR:1. 41; 95% CI: 1.19–1.67) as well as age groups <70 (OR: 1.46; 95% CI: 1.13–1.87) and 71–80 (OR: 1 1.59; 95% CI: 1.29–1.97), but not in the age group 80+ (OR: 1.03; 95% CI: 0.82–1.29) (Table 3). The analysis revealed that an HbA1c value of ≥8.5% was associated with an increased risk of pancreatic cancer. However, this association was significant only in the male patients, with an OR of 1.45 (95% CI: 1.10–1.91) (Table 3).

### 3.3. Association between Fasting Glucose Values and Subsequent Pancreatic Cancer Diagnosis

The average fasting glucose value was higher in the cases (143.9 mg/dL; SD: 46.3) than in the controls (135.1 mg/dL; SD: 50.5). Only the severely elevated average fasting glucose values prior to the index date were significantly associated with an increased risk of subsequent pancreatic cancer (OR: 1.38; 95% CI: 1.22–1.57). However, this association was significant only in the male patients, with an OR of 1.54 (95% CI: 1.20–1.97) and in the age group <70 years (OR: 1.96; 95% CI: 1.36–2.82) (Table 3).

### 3.4. Association between Antihyperglycemic Therapy and Subsequent Pancreatic Cancer Diagnosis

Sodium–glucose cotransporter 2 (SGLT 2)—inhibitor was the only antihyperglycemic therapy that was negatively associated with pancreatic cancer risk in the total study population (OR: 0.80; 95% CI: 0.74–0.87 per therapy year) as well as in the age- and sex-stratified subgroups. This negative association was stronger in women (OR: 0.66; 95% CI: 0.55–0.78) than in men (OR: 0.85; 95% CI: 0.78–0.93), and similar in the three age groups (ORs from 0.73 to 0.81). Metformin was weakly associated with increased pancreatic cancer risk (OR: 1.05; 95% CI: 1.02–1.08); however, this association can be considered non-clinically relevant.

## 4. Discussion

We conducted a retrospective case–control study using a large database with electronic medical records to investigate the association between serum HbA1c levels, antihyperglycaemic therapy, and the subsequent diagnosis of pancreatic cancer in patients with T2D. Elevated average HbA1c levels in patients with T2D were associated with an increased subsequent diagnosis of pancreatic cancer. Notably, our data are consistent with recent findings, e.g., by Lemanska et al., showing that a 1 mmol/mol increase in HbA1c prior to initial diabetes diagnosis was associated with higher odds ratios for pancreatic cancer [12].

The age- and sex-stratified analyses showed that average HbA1c levels between 6.5 and 8.4% were significantly associated with increased pancreatic cancer risk in individuals aged <70 and 71–80 years. This is notable as the average age at the first diagnosis of pancreatic cancer is 71 years [13]. It is noteworthy that this effect was not observed in the over-80 age group, which lends support to the prevailing cautious therapeutic approach of antihyperglycemic therapy in elderly diabetics. This approach is designed to avoid hypoglycemia, which carries particular risks. These include an increased risk of falls and the potential development of dementia. In turn, dementia has a negative impact on memory and motor skills [14,15]. Furthermore, it increases susceptibility to cardiovascular disease [16]. Given that the highest incidence of pancreatic cancer is found in the 80+ age group [17], these results suggest that HbA1c alone cannot be the sole cause of pancreatic cancer development. Rather, other factors must play a role. SGLT2 inhibitors were the only antihyperglycaemic therapy negatively associated with pancreatic cancer risk in the overall study population (OR: 0.80; 95% CI: 0.74–0.87 per year of therapy) and in the age- and sex-stratified subgroups. These findings are in line with the research by Ren et al. who showed that SGLT2 promotes pancreatic cancer progression via the hnRNPK-YAP1 axis in the Hippo signaling pathway [18], and by Dutka et al. who suggested that the ability of SGLT2 inhibitors to block glucose uptake by cancer cells may be a therapeutic approach given the metabolic reprogramming in cancer cells [19].

This negative association was more pronounced in women (OR: 0.66; 95% CI: 0.55–0.78) than in men (OR: 0.85; 95% CI: 0.78–0.93) and was consistent across all the age groups (ORs from 0.73 to 0.81). The exact reasons for these gender differences remain unclear. In contrast to other studies, metformin use was only weakly associated with an increased risk of pancreatic cancer [20,21]. The meta-analysis by Zhang et al. on metformin therapy and pancreatic cancer development, which included 37 studies, showed a significantly reduced relative risk of pancreatic cancer incidence and mortality in patients on metformin therapy [22]. Therefore, our results should be interpreted with caution, taking into account the context and significance level.

It is important to note that our results have limitations, such as unrecorded lifestyle variables which may differ between subjects. Many factors influence the development of pancreatic cancer, including known risks such as diabetes, alcohol consumption, smoking, body mass index (BMI), and pancreatitis, as well as factors such as allergies, immune status, family history, microbiota, and environmental factors. For example, a correlation has been found between the likelihood of developing pancreatic cancer and the region of residence [23]. In addition, lifestyle changes such as smoking cessation can significantly reduce the risk of pancreatic cancer to the level of non-smokers within 10–20 years [24]. The literature also indicates that attendance at preventive medical examinations, such as those conducted by dentists for periodontitis treatment, may exert an influence on the development of pancreatic cancer [25,26].

In conclusion, our data show a significant association between the average HbA1c levels and the subsequent diagnosis of pancreatic cancer. SGLT-2 inhibitors were the only antihyperglycemic therapy negatively associated with pancreatic cancer risk in the entire study population. Our results suggest that it is not only the level of HbA1c or its reduction that predicts the development of pancreatic cancer. Other factors must also play an important role. However, further studies are needed to better understand the exact relationships and possibly identify a new approach to the early risk stratification of patients at risk of developing pancreatic cancer.

## Figures and Tables

**Table 1:** Patient characteristics.

Variable	Pancreatic Cancer (*n* = 1682)	No Pancreatic Cancer (*n* = 8410)	SMD
Age (in years)			
Mean (SD)	74.4 (9.4)	75.0 (9.9)	−0.01
<70	478 (28.4)	2306 (27.4)	
70–79	650 (38.6)	3051 (36.3)	
80+	554 (33.0)	3053 (36.3)	
Sex			
Female	793 (47.2)	3975 (47.3)	−0.01
Male	889 (52.8)	4435 (52.7)	
Diabetes duration (years), mean (SD)	6.8 (4.5)	6.8 (4.6)	0.01
Conditions documented within five years prior to the index date			
Obesity	383 (22.8)	1818 (21.6)	−0.01
Hypertension	1418 (84.3)	7264 (86.4)	0.02
Lipid metabolism disorder	994 (59.1)	5040 (59.9)	0.01
Pancreas diseases	1,284 (16.9)	1336 (15.9)	0.01
Diseases of the esophagus, stomach, and duodenum	910 (54.1)	4925 (53.8)	0.00
Chronic kidney disease	329 (19.6)	1580 (18.8)	−0.01

Data are the absolute samples and percentages unless otherwise specified. SD = standard deviation; SDM = standardized mean difference.

**Table 2:** Descriptive analysis of outcomes (HbA1c values and antihyperglycemic therapy).

Variable	Pancreatic Cancer (*n* = 1682)	No Pancreatic Cancer (*n* = 8410)	*p* Value
Number of documented HbA1c values; mean (SD)	14.5 (12.2)	13.5 (12.2)	<0.001
HbA1c (%); mean (SD)	7.0 (1.1)	6.8 (1.1)	<0.001
Average HbA1c < 6.5% (N, %)	940 (55.9)	5438 (64.6)	<0.001
Average HbA1c 6.5–8.4% (N, %)	585 (34.8)	2345 (27.9)	
Average HbA1c ≥ 8.5% (N, %)	157 (9.3)	627 (7.5)	
Fasting glucose (mg/dL); mean (SD) (documented in 1440 cases and 7108 controls)	143.9 (46.3)	135.1 (50.5)	<0.001
Average fasting glucose <125 mg/dL (N, %)	602 (41.8)	3619 (50.9)	<0.001
Average fasting glucose 125–150 mg/dL (N, %)	343 (23.8)	1657 (23.3)	
Average fasting glucose >150 mg/dL (N, %)	495 (34.4)	1832 (25.8)	
Metformin (N, %)	927 (56.9)	4053 (48.2)	<0.001
Sulfonylureas (N, %)	293 (17.4)	1014 (21.1)	<0.001
DPP-4i (N, %)	465 (27.7)	1848 (22.0)	<0.001
GLP-1RA (N, %)	52 (3.1)	284 (3.4)	0.404
SGLT-2 (N, %)	161 (9.6)	1011 (12.0)	<0.001
Insulin (N, %)	558 (33.2)	1921 (22.8)	<0.001

Data are the absolute samples and percentages unless otherwise specified. The patients can receive different drug classes during the follow-up period. SD = standard deviation.

**Table 3:** Association between HbA1c values, fasting glucose values, antihyperglycemic therapy, and subsequent pancreatic cancer diagnosis.

	OR (95% CI)					
Variable	Total	Women	Men	Age < 70	Age 71–80	Age 80+
HbA1c 6.5–8.4% vs. <6.5%	1.38 (1.22–1.57) *	1.31 (1.07–1.61) *	1.41 (1.19–1.67) *	1.46 (1.13–1.87) *	1.59 (1.29–1.97) *	1.03 (0.82–1.29)
HbA1c ≥ 8.5% vs. <6.5%	1.41 (1.16–1.73) *	1.32 (0.96–1.82)	1.45 (1.10–1.91) *	1.41 (1.00–1.98)	1.39 (0.97–2.00)	1.28 (0.84–1.95)
Fasting glucose 125–150 mg/dL vs. <125 mg/dL	1.15 (0.98–1.35)	1.30 (1.02–1.65)	1.16 (0.92–1.45)	1.45 (1.05–2.01)	1.05 (0.80–1.39)	1.03 (0.78–1.36)
Fasting glucose >150 mg/dL vs. <125 mg/dL	1.38 (1.16–1.65) *	1.21 (0.91–1.60)	1.54 (1.20–1.97) *	1.96 (1.36–2.82) *	1.46 (1.07–1.99)	1.13 (0.82–1.55)
Metformin (effect per therapy year)	1.05 (1.02–1.08) *	1.04 (1.00–1.08)	1.06 (1.02–1.11) *	1.08 (1.02–1.14)	1.03 (0.98–1.08)	1.06 (1.01–1.11)
Sulfonylureas (effect per therapy year)	1.05 (1.02–1.09) *	1.06 (1.00–1.12)	1.06 (1.01–1.12)	1.07 (0.98–1.18)	1.07 (1.00–1.13)	1.07 (1.01–1.13)
DPP-4i (effect per therapy year)	1.00 (0.96–1.03)	1.02 (0.97–1.08)	0.99 (0.94–1.04)	0.97 (0.90–1.05)	1.01 (0.95–1.07)	1.02 (0.96–1.08)
GLP-1RA (effect per therapy year)	0.92 (0.81–1.04)	0.90 (0.72–1.13)	0.94 (0.80–1.11)	0.87 (0.71–1.08)	0.86 (0.69–1.07)	0.97 (0.63–1.48)
SGLT-2 (effect per therapy year)	0.80 (0.74–0.87) *	0.66 (0.55–0.78) *	0.85 (0.78–0.93) *	0.81 (0.71–0.92) *	0.79 (0.69–0.89) *	0.73 (0.59–0.91) *
Insulin (effect per therapy year)	1.02 (0.99–1.05)	1.03 (0.98–1.08)	1.01 (0.97–1.06)	1.05 (0.99–1.13)	1.02 (0.97–1.08)	1.02 (0.97–1.08)

Abbreviations: OR—odds ratio; CI—confidence interval. * < 0.01.

## Data Availability

The data presented in this study are available on request from the corresponding author.
